# New Dominant-Negative IL6ST Variants Expand the Immunological and Clinical Spectrum of GP130-Dependent Hyper-IgE Syndrome

**DOI:** 10.1007/s10875-023-01517-4

**Published:** 2023-06-05

**Authors:** Tiphaine Arlabosse, Marie Materna, Orbicia Riccio, Caroline Schnider, Federica Angelini, Matthieu Perreau, Isabelle Rochat, Andrea Superti-Furga, Belinda Campos-Xavier, Sébastien Héritier, Anaïs Pereira, Caroline Deswarte, Romain Lévy, Marco Distefano, Jacinta Bustamante, Marie Roelens, Raphaël Borie, Mathilde Le Brun, Bruno Crestani, Jean-Laurent Casanova, Anne Puel, Michaël Hofer, Claire Fieschi, Katerina Theodoropoulou, Vivien Béziat, Fabio Candotti

**Affiliations:** 1 Pediatric Immuno-Rheumatology of Western Switzerland, Pediatrics Service, Women-Mother-Child Department, Lausanne University Hospital, Lausanne, Switzerland; 2 Laboratory of Human Genetics of Infectious Diseases, Necker Branch, Institut National de La Santé Et de La Recherche Médicale (INSERM), U1163 Paris, France; 3 Paris Cité University, Imagine Institute, Paris, France; 4 Division of Immunology and Allergy, Lausanne University Hospital and University of Lausanne, Lausanne, Switzerland; 5 Pediatric Pulmonology and Cystic Fibrosis Unit, Pediatrics Service, Women-Mother-Child Department, Lausanne University Hospital, Lausanne, Switzerland; 6 Division of Genetic Medicine, Lausanne University Hospital and University of Lausanne, Lausanne, Switzerland; 7 Division of Pediatric Hematology and Oncology, Armand Trousseau Hospital, Sorbonne University, Paris, France; 8 St. Giles Laboratory of Human Genetics of Infectious Diseases, Rockefeller Branch, The Rockefeller University, New York, NY USA; 9 Study Center for Primary Immunodeficiencies, AP-HP, Necker Children Hospital, Paris, France; 10 Department of Medicine, Bichat Hospital, AP-HP, Paris, France; 11 Department of Pulmonology A, Reference Center for Rare Pulmonary Diseases, Bichat Hospital, AP-HP, Paris, France; 12 Department of Pediatrics, Necker Hospital for Sick Children, AP-HP, 75015 Paris, France; 13 Howard Hughes Medical Institute, The Rockefeller University, New York, NY 10065 USA; 14 Department of Clinical Immunology, Paris Cité University, Assistance Publique Hôpitaux de Paris (AP-HP), Saint-Louis Hospital, Paris, France

**Keywords:** Hyper-IgE, Job's syndrome, IL6ST, STAT3, Inborn errors of immunity, GP130

## Abstract

Patients with autosomal dominant (AD) hyper-IgE syndrome (HIES) suffer from a constellation of manifestations including recurrent bacterial and fungal infections, severe atopy, and skeletal abnormalities. This condition is typically caused by monoallelic dominant-negative (DN) *STAT3* variants. In 2020, we described 12 patients from eight kindreds with DN *IL6ST* variants resulting in a new form of AD HIES. These variants encoded truncated GP130 receptors, with intact extracellular and transmembrane domains, but lacking the intracellular recycling motif and the four STAT3-binding residues, resulting in an inability to recycle and activate STAT3. We report here two new DN variants of *IL6ST* in three unrelated families with HIES-AD. The biochemical and clinical impacts of these variants are different from those of the previously reported variants. The p.(Ser731Val*f*s*8) variant, identified in seven patients from two families, lacks the recycling motif and all the STAT3-binding residues, but its levels on the cell surface are only slightly increased and it underlies mild biological phenotypes with variable clinical expressivity. The p.(Arg768*) variant, identified in a single patient, lacks the recycling motif and the three most distal STAT3-binding residues. This variant accumulates at the cell surface and underlies severe biological and clinical phenotypes. The p.(Ser731Val*f*s*8) variant shows that a DN GP130 expressed at near normal levels on the cell surface can underlie heterogeneous clinical presentations, ranging from mild to severe. The p.(Arg768*) variant demonstrates that a truncated GP130 protein retaining one STAT3-binding residue can underlie severe HIES.

**Supplementary Information:**

The online version contains supplementary material available at 10.1007/s10875-023-01517-4.

## Introduction

Hyper-IgE syndrome (HIES) is an inborn error of immunity (IEI) characterized by recurrent infections (e.g., staphylococcal skin abscesses, bacterial pneumonia, chronic mucocutaneous candidiasis), severe atopy, connective tissue abnormalities (e.g., osteopenia, spontaneous fractures, primary tooth retention, joint hyperextensibility), high serum immunoglobulin E (IgE) concentration, eosinophilia, and an impaired inflammatory response [1–5]. Since the first description of HIES patients by Davis et al. in 1966, three genes (*STAT3*, *ZNF341*, and *IL6ST*) have been implicated in this disease [5–12]. These three genes underlie four allelic forms of the disease: two autosomal dominant (AD) forms of HIES due to dominant-negative (DN), loss-of-function (LOF) variants of *STAT3* or *IL6ST*, and two autosomal recessive (AR) forms of HIES due to biallelic LOF variants of *ZNF341*, or biallelic hypomorphic variants of *IL6ST* [5, 6, 8, 10, 12–15]. Most cases of HIES are due to DN *STAT3* variants. *STAT3* expression is controlled by* ZNF341*, accounting for the similarities between the genetic diseases caused by defects of these two genes [9]. STAT3 is a pleiotropic transcription factor crucial for the signaling pathways of multiple cytokine receptors [5]. Complete Stat3 deficiency is embryo-lethal in the mouse model [16]. Our understanding of the molecular mechanisms underlying HIES was improved by the discovery of causal variants of genes encoding proteins involved in signaling by members of the IL-6 family of cytokines. This family includes IL-6, IL-11, IL-27, oncostatin M (OSM), ciliary neurotrophic factor (CNTF), cardiotrophin 1 (CT-1), cardiotrophin-like cytokine (CLC), and leukemia inhibitory factor (LIF) [16, 17]. All these cytokines share a common coreceptor, GP130, encoded by *IL6ST*. Binding to this receptor induces STAT3 signaling. Complete GP130 deficiency underlies lethal Stüve-Wiedemann syndrome [11]. By contrast, AR and AD HIES caused by DN or biallelic partial LOF variants of *IL6ST* preferentially impair IL-6 and IL-11 signaling [6, 15]. In addition, complete IL6R and IL11RA deficiencies (or biallelic pathogenic variants of *IL6ST* impairing only IL-11 signaling) underlie most of the immunological and extra-hematopoietic symptoms, respectively, of HIES patients [18–20]. Careful investigations of HIES phenotypes have, thus, revealed the major contribution of defects of IL-6 and IL-11 signaling via STAT3 to the pathophysiology of the disease.

The first 12 patients with AD HIES due to monoallelic pathogenic variants of *IL6ST* were reported in 2020 [6]. No other patients have been reported at the time of this writing. All the causal variants identified concerned sites C-terminal to the transmembrane domain and N-terminal to the four STAT3-binding residues and the recycling motif, resulting in mutant proteins able to migrate to and accumulate at the cell surface (due to the lack of the recycling motif), but unable to activate the JAK-STAT3 pathway when engaged by IL-6 family cytokines. Among the 12 patients described, the kindred with the mildest phenotype had the most distal variant (p.(Thr761Ilefs*29)), removing all four STAT3-binding sites and the recycling motif, but resulting in retention of the SHP2/SOCS3 binding site (Tyr759), a motif required for GP130-mediated activation of the MAPK/ERK pathway [21]. The GP130 variants reported in the population-based Genome Aggregation Database (gnomAD) affecting residues C-terminal to the recycling motif and one or more of the STAT3-binding residues resulted in milder functional defects when characterized in vitro. Variants C-terminal to the Tyr759 SHP/SOCS3 binding site and N-terminal to the recycling motif were predicted to be associated with a milder HIES phenotype [6]. We report here two new DN *IL6ST* variants identified in patients with a clinical presentation of AD HIES, including one C-terminal to the Tyr759 SHP/SOCS3 binding site and to the first STAT3 binding site, but N-terminal to the recycling motif.

## Methods

### Genetic Analysis

For P2 (kindred A) and P5 (kindred B), we analyzed a panel of 84 genes implicated in monogenic human inborn errors of innate immunity. Genomic DNA was extracted from a whole-blood sample collected in a tube containing EDTA. The regions captured with hybridization libraries (Agilent Technologies) targeting the exonic and flanking intronic regions (± 20 bp) were sequenced on an Illumina NextSeq500. The sequences obtained were demultiplexed and aligned to the hg18 reference human genome (BWA). The mean depth of coverage was 1500 × . Data were analyzed with the Genome Analysis Toolkit (GATK; Haplotypecaller, Unifigenotyper, Samtools, and Freebayes) according to Broad Institute best practice rules. All relatives of the probands of kindreds A (P2) and B (P5) underwent genetic testing by Sanger sequencing, which identified P1, P3, P4, P6, and P7.

For P8, whole-exome sequencing data were generated by next-generation sequencing (NGS) methods on an Illumina NextSeq500 platform. Genomic DNA was extracted from peripheral blood leukocytes according to standard procedures. Exome libraries (Twist Bioscience Reagent Kit) were sequenced on a NextSeq500 (Illumina). Raw reads were mapped to the human reference genome (hg19/GRCh37). Sequenced data were further annotated with ALISSA Interpret (Agilent Technologies), using the *.vcf (VCF, variant call format) files generated by an in-house strategy, and filtered against a panel of genes known to be associated with inborn errors of immunity in humans. Familial segregation of the variant was investigated by Sanger sequencing.

### Cell Culture

GP130-deficient human embryonic kidney (HEK) 293 T cells were cultured in DMEM supplemented with 10% fetal bovine serum (Sigma-Aldrich), as previously described [6]. PBMCs were obtained from whole-blood samples from healthy volunteers and the patients by Ficoll-Hypaque centrifugation (Amersham-Pharmacia-Biotech).

### Plasmids and Transfection for Overexpression Experiments

The human *IL6ST* expression vectors and the C-terminal Myc/DDK-tagged pCMV6 empty vector (EV) were purchased from OriGene. Constructs carrying the c.2155dupA, c.2190dupG, and 2302A > T *IL6ST* mutant alleles were generated by direct mutagenesis with the QuikChange II XL Site-Directed Mutagenesis Kit according to the manufacturer’s instructions (Agilent Technologies). GP130-deficient HEK293T cells were transiently transfected with the various constructs. For overexpression experiments, GP130-deficient HEK293T cells were transfected with pCMV6-GP130 vector (WT or mutant) or with an empty pCMV6 vector with the X-tremeGENE 9 DNA Transfection Reagent (Roche), as previously described [6], and cultured for 24 h, 48 h, or 72 h before being harvested.

### Flow Cytometry

For overexpression, transfected HEK293T cells were washed with FACS buffer (PBS 1X + 2% FBS + 2 mM EDTA) and stained by incubation for 1 h at room temperature with a PE-conjugated mouse anti-human CD130 antibody (AM64; BD; 1:50) and the Aqua Live/Dead Cell Stain Kit (Thermo Fisher Scientific) in the same buffer. For PBMC staining, we incubated two million cells for 30 min at room temperature with the antibodies listed in Table S1 in FACS buffer. Data were acquired with a Gallios flow cytometer (Beckman Coulter) and analyzed with FlowJo v.10.5.3 software.

### Immunoblotting

Total protein extracts were prepared by mixing transfected HEK293T cells with lysis buffer (50 mM Tris, pH 7.4, 150 mM NaCl, 2 mM EDTA, and 0.5% Triton X-100) and incubating for 30 min at 4 °C. A mixture of protease inhibitors and phosphatase inhibitors was added to the buffers before use: aprotinin (10 µg/mL; Sigma-Aldrich), PMSF (1 mM; Sigma-Aldrich), leupeptin (10 µg/mL; Sigma-Aldrich), protease inhibitor cocktail (1 × ; Sigma-Aldrich). The lysates were centrifuged for 15 min at 16,000 × *g*, and the supernatant was collected for immunoblotting. For each sample, 30 µg protein extract was separated by SDS-PAGE and immunoblotting was performed with antibodies against GP130 (E-8; Santa Cruz) and GAPDH (FL335; Santa Cruz).

### Cytokines

IL-6, IL-11, LIF, and OSM were purchased from Miltenyi Biotec. IL-27 was purchased from Bio-Techne, IL-6/IL-6R alpha was purchased from R&D Systems, and IFN-α (Intron A) was purchased from Merck. All cytokines were used at a final concentration of 100 ng/mL, with the exception of IFN-α, which was used at 10^5^ U/mL.

### Phospho-Flow Cytometry

The levels of p-STAT3 and p-STAT1 in GP130-deficient HEK293T cells were evaluated 48 h after transfection with pCMV6-GP130 (WT or mutant) or with an empty pCMV6 vector. After 15 min of stimulation with the various cytokines, cells were fixed with fix buffer I (1:1 volume; BD Biosciences) for 10 min at 37 °C. Cells were then washed with FACS buffer (1X PBS + 2% FBS + 2 mM EDTA) and permeabilized by incubation for 20 min at 4 °C in Perm buffer III (BD Biosciences). Finally, cells were stained by incubation for 1 h at 4 °C with anti-STAT3-pY705 (antibody dilution 4:50; BD; 557815), or anti-STAT1-pY701 (antibody dilution 4:50; BD; 612597). Cells were analyzed on a NovoCyte Flow cytometer (Agilent).

### RNA Purification and RT-qPCR

Total RNA was extracted from the indicated cells using the RNeasy Extraction Kit (Qiagen). RNA was reverse-transcribed with SuperScript II reverse transcriptase (Thermo Fisher Scientific) and oligo-dT primers (Thermo Fisher Scientific). Quantitative PCR was then performed with the Applied Biosystems Assays-on-Demand probes/primers specific for IL6ST-FAM (Hs00174360_m1). The data were normalized against the expression (ΔCt) of GUS (13-glucuronidase-VIC, 4326320E) and are displayed as 2^–ΔCt^.

### Luciferase Reporter Assay

GP130-deficient HEK293T cells were cultured in DMEM supplemented with 10% FCS in 96-well plates and transfected with the pGL4.47{luc2P/SIE/Hygro} (Promega) reporter plasmids (100 ng/well), the pRL-SV40 vector (10 ng/well), and the WT or mutant pCMV6-GP130 constructs (100 ng/well) in the presence of X-tremeGENE 9 DNA Transfection Reagent (Roche). The DN effect of the p.(Ser731Valfs*8) and p.(Arg768*) mutant alleles was assessed by transfecting GP130-deficient HEK293T cells with the pGL4.47{luc2P/SIE/Hygro} (Promega) reporter plasmids (100 ng/well for a 96-well plate), the pRL-SV40 vector (10 ng/well), the WT pCMV6-GP130 (25 ng/well), and various concentrations of the mutant pCMV6-GP130 constructs (12.5 ng/well, 25 ng/well, 50 ng/well, 100 ng/well, or 200 ng/well) in the presence of X-tremeGENE 9 DNA Transfection Reagent (Roche). We ensured that the same amount of DNA was added to each well for transfection, by adding various amounts of empty pCMV6 vector to make up the total amount of DNA present to 335 ng DNA/well. Cells transfected with the pGL4.47{luc2P/SIE/Hygro} (Promega) reporter plasmids, the pRL-SV40 vector, and the WT pCMV6-GP130 only or the indicated mutant pCMV6-GP130 only or the empty pCMV6 were also included as controls. After 24 h of transfection, the medium was removed and cells were cultured with fresh DMEM supplemented with 10% FCS and the cytokines indicated for an additional 24 h before being used for luciferase assays with the Dual-Glo luciferase assay system (Promega), as previously described [6]. The promoter activity of each well was assessed by determining the ratio of firefly luciferase activity to *Renilla* luciferase activity. Technical triplicates were performed for each experiment.

### Mass Cytometry on Fresh Whole Blood

Whole-blood mass cytometry was performed on 200 µL fresh blood with a customized antibody panel (Table S2), in accordance with Fluidigm recommendations. Labeled cells were subjected to dead cell staining overnight, and were then frozen and stored at − 80 °C until use. Acquisition was performed on a Helios machine (Fluidigm) and the data were analyzed with OMIQ software.

## Results

### Case Reports

We studied eight patients from three unrelated kindreds. Clinical summaries for these patients are provided in Table 1.

**Table 1:** Summary of the clinical characteristics of the patients with AD *IL6ST* variants

	Kindred A		Kindred B					Kindred C	Published patients
	P1	P2	P3	P4	P5	P6	P7	P8	(*n* = 12)
Mutant allele	p.Ser731Val*fs* *8		p.Ser731Val*fs* *8					p.Arg768*	Various
Age (years)	38	11	70	47	45	33	41	20	Range = 12-72
Sex	M	F	F	F	F	F	M	M	8M, 4F
HIES score	37	28	13	12	42	24	ND	51	Range = 20-70
IgE levels (IU/mL)	1393	10,600	202	43	8290	330	ND	3258	Mean = 6057 Range = 218–18,700
High IgE levels	+	+	+	-	+	+	ND	+	100%
Eosinophilia	-	+	-	-	+	-	ND	+	73%
Atopic dermatitis	+	-	-	-	+	+	-	+	73%
Asthma	-	+	+	-	+	+	-	+	67%
Allergy									
Food allergies	-	-	-	-	+	-	-	-	9%
Respiratory allergies	-	+	-	-	+	+	-	+	64%
Skin abscesses	+	-	-	-	-	-	-	-	36%
Chronic mucocutaneous candidiasis (CMC)	-	-	-	-	-	-	-	-	9%
Recurrent skin infections (other than CMC)	+	-	-	-	-	-	-	-	25%
Severe chicken pox/shingles	-	-	-	-	-	-	-	-	27%
Upper respiratory tract infections	+	+	+	-	+	+	-	+	55%
Pulmonary disease									
Recurrent infections	+	+	+	-	+	-	-	+	92%
Pneumonia	+	-	+	-	+	+	-	+	91%
Bronchiectasis	ND	+	+	-	+	+	-	+	60%
Pneumatocele	ND	-	ND	-	+	-	-	-	55%
Fatal infection	-	-	-	-	-	-	-	-	17%
Connective tissue abnormalities									
Characteristic face	-	-	-	-	-	-	-	+	45%
High palate	-	-	-	-	-	-	-	-	27%
Scoliosis	+	+	+	+	+	+	+	-	64%
Joint hyperextensibility	+	+	ND	-	-	-	-	+	55%
Bone fractures with minimal trauma	+	-	-	+	-	-	-	+	36%
Clubfoot	-	-	-	-	-	-	-	-	18%
Supernumerary teeth	-	-	-	-	-	-	-	-	30%
Deciduous tooth retention	+	+	-	-	-	-	-	+	82%

#### Kindred A

P1 is a 38-year-old French man. He was born to non-consanguineous parents and had a history of mild eczema during childhood and recurrent skin abscesses. He also experienced recurrent fractures (foot, rib, scaphoid), a shoulder dislocation, and had moderate scoliosis in addition to the retention of deciduous teeth. At the age of 31 years, he suffered from pneumonia with pleural effusion complicated by pneumothorax and requiring chest drainage. His IgE levels were 1393 IU/mL and his National Institutes of Health (NIH) HIES score was 37.

**Fig. 1:**
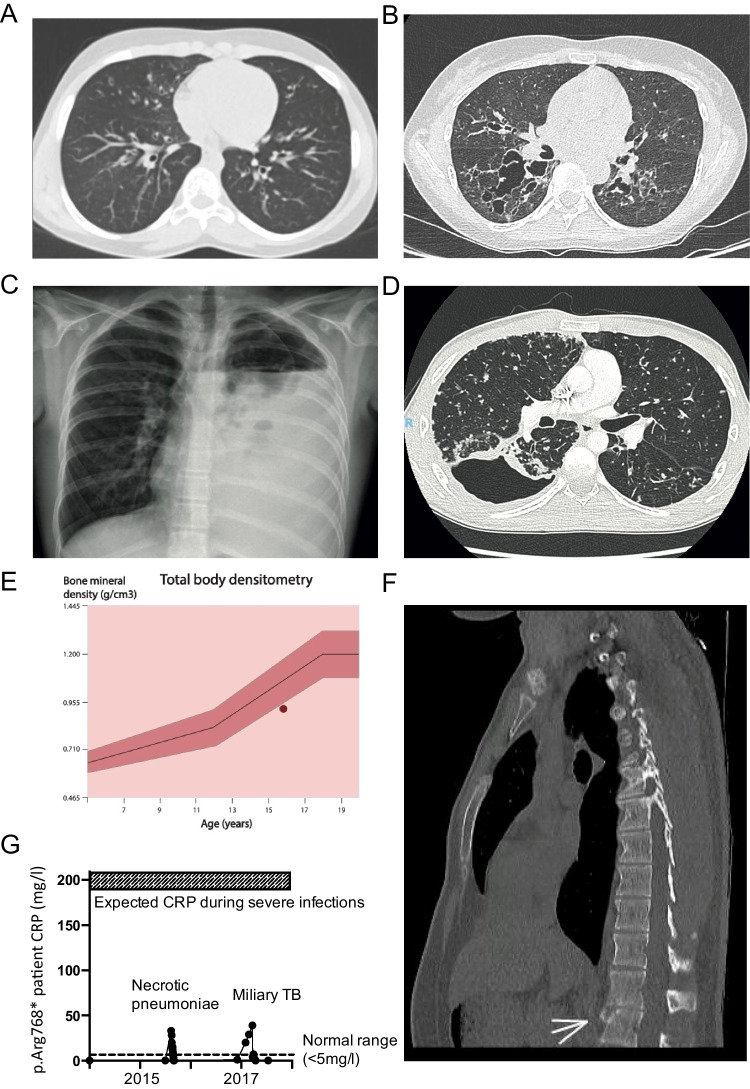
Clinical features of patients P2 (**A**), P5 (**B**), and P8 (**C**–**G**). **A** Chest CT scan of patient P2 showing bronchiectasis. **B** Chest CT scan of patient P5 showing pneumatocele. **C** Chest X-ray showing parapneumonic effusion and pneumothorax during severe MRSA and *S. pneumoniae* necrotizing pneumonia in patient P8 at the age of 13 years. **D** Chest CT scan showing bronchopleural fistula during miliary tuberculosis in patient P8 at the age of 15 years. **E** Total body densitometry showing a bone density below the normal range in patient P8. **F** Chest CT scan showing signs of an old fracture at L1 in patient P8 at the age of 21 years. **G** Impaired acute-phase response with a maximum CRP concentration of 33–39 mg/L during episodes of necrotizing pneumonia and miliary tuberculosis in patient P8

P2 is the 11-year-old daughter of P1. Her parents are French and non-consanguineous. She presented numerous episodes of acute otitis media in early childhood and then suffered from atypical asthma poorly controlled by nebulization therapy and persistent cough. Chest computed tomography (CT) showed bilateral bronchiectasis without pneumatoceles (Fig. 1A). P2 had retentional acne beginning at puberty, but no history of skin abscesses. She was vaccinated with bacillus Calmette-Guérin (BCG) without complications. She presented deciduous tooth retention, which was treated surgically, and had moderate scoliosis and hyperextensibility but no facial dysmorphism. P2 had eosinophilia (1.8 × 10^9^/L) and IgE levels of 10,600 IU/mL. She had normal serum total IgG and IgG subclass levels and normal antibody responses to both nonconjugated and conjugated vaccines. The NIH HIES score of P2 was 28.

#### Kindred B

P3 is a 70-year-old Moroccan woman with a medical history of asthma treated with steroids in her thirties and a pulmonary abscess requiring surgical lobectomy at the age of 60 years. She had a serum IgE concentration of 202 IU/mL and her NIH HIES score was 13. GP130 deficiency was confirmed in P3 following the genetic diagnosis of her daughter (P5).

P4 is a 47-year-old woman born to non-consanguineous Moroccan parents. She is the daughter of P3 and sister of P5, P6, and P7. She had a significant medical history of severe scoliosis (Cobb angle 54°) requiring surgical treatment at the age of 17 years and osteoporosis diagnosed following the observation of delayed healing for collarbone and costal fractures suffered in a motorcycle accident. She also had a slowly healing traumatic wrist fracture at the age of 56 years. She had no respiratory symptoms, no eczema, and no skin abscesses. She had an eosinophil count of 0.18 × 10^9^/L and an IgE concentration of 42 IU/mL. Her NIH HIES score was 12. GP130 deficiency was confirmed in P4 during familial segregation analysis following the diagnosis of her sister (P5).

P5 is a 45-year-old woman. Her parents are Moroccan and non-consanguineous and she is the daughter of P3 and sister of P4, P6, and P7. P5 had pneumonia at the ages of 3 and 4 years and suffered repeated lower respiratory tract infections from the age of 33 years, for which the following causal pathogens were identified: *Staphylococcus aureus*, *Haemophilus influenzae*, *Streptococcus pneumoniae*, *Achromobacter* sp., *Pseudomonas aeruginosa*, *Stenotrophomonas maltophilia*, *Achromobacter xylosoxidans*, *Mycobacterium avium*, and *Mycobacterium abscessus*. During the last episode, pneumatocele was observed on the CT scan (Fig. 1B). At the age of 43 years, P5 had a maxillary aspergilloma that required surgery and a herpes zoster infection of the right arm. She had no skin abscesses, but suffered several episodes of allergic bronchopulmonary aspergillosis (ABPA) treated with antifungal therapy, steroids, and omalizumab, a monoclonal antibody that selectively binds to IgE. This treatment was used before the patient was diagnosed with HIES, and did not improve her ABPA. P5 presented no apparent connective tissue abnormalities other than mild scoliosis. She displayed marked eosinophilia (4.04 × 10^9^/L) and high IgE levels (8290 IU/mL). The NIH score of P5 was 42.

P6 is the youngest daughter of P3 (33 years old), and the sister of P4, P5, and P7. She had a medical history of moderate scoliosis, childhood asthma, multiple allergies (grass, dust mites), and urticaria effectively treated by subcutaneous immunotherapy during childhood. She suffered from chronic cough and had a *Streptococcus pneumoniae* lung infection at age 33. She had moderate, but diffuse bronchiectasis, with an eosinophil count of 0.5 × 10^9^/L and an IgE level of 330 IU/mL. Her NIH HIES score was 24. She was also confirmed to have IL6ST deficiency on the basis of familial segregation analysis. P6 has a daughter and a son, and neither of whom carries the *IL6ST* variant present in the kindred.

P7 is the 41-year-old son of P3. He had a medical history of scoliosis not requiring surgical treatment. He had no history of asthma or allergy. He recently had a *Helicobacter pylori* infection, which was successfully treated with antibiotics. He has a 9-year-old son who is currently asymptomatic and has not been tested for the *IL6ST* variant. It was not possible to calculate the HIES score of P7.

#### Kindred C

P8 is a 20-year-old man, the only son from a non-consanguineous union between a Colombian mother and a Swiss father, with no family history of immunodeficiency. He has suffered from severe eczema and severe asthma since childhood, treated according to step 4 of the GINA guidelines. At the age of 13 years, he was referred for immunodeficiency screening following an episode of necrotizing pneumonia with parapneumonic effusion caused by methicillin-resistant *Staphylococcus aureus* and *Streptococcus pneumoniae* (Fig. 1C). At the age of 15 years, he was hospitalized for miliary tuberculosis complicated by a bronchopleural fistula (Fig. 1D) in the absence of prior BCG vaccination. P8 had no history of cold skin abscesses, or significant fungal infections. In addition to his infectious complications, he presented a delayed loss of primary teeth, in the absence of other oral abnormalities. At the age of 19 years, treatment with dupilumab, a monoclonal antibody blocking IL-4 and IL-13 signaling, was initiated, leading to a significant improvement of the patient’s eczema, but no effects on asthma control. P8 has had no relevant infections since treatment initiation. Bone densitometry revealed osteopenia (Fig. 1E) and a recent chest CT scan revealed an old vertebral fracture at L1 level of which the patient was unaware (Fig. 1F). Physical examination revealed a large nasal bridge, prominent forehead, and hyperextensible elbows and knees. The immunological features of the patient are reported in Table S3 and include eosinophilia (maximum value 6.36 × 10^9^/L), high IgE levels (up to 3258 IU/L), low levels of T and B memory cells, Th17 cell levels within the normal range, and unusually low levels of inflammation markers during severe infection (e.g., C-reactive protein at 39 mg/L during miliary tuberculosis and 33 mg/L during necrotizing pneumonia) (Fig. 1G). Dupilumab treatment lowered total IgE levels (from 1938 to 1011 kU/L in 4 months). The NIH HIES score of P8 was 51.

### Identification of Two New *IL6ST* Variants Causing AD HIES

**Fig. 2:**
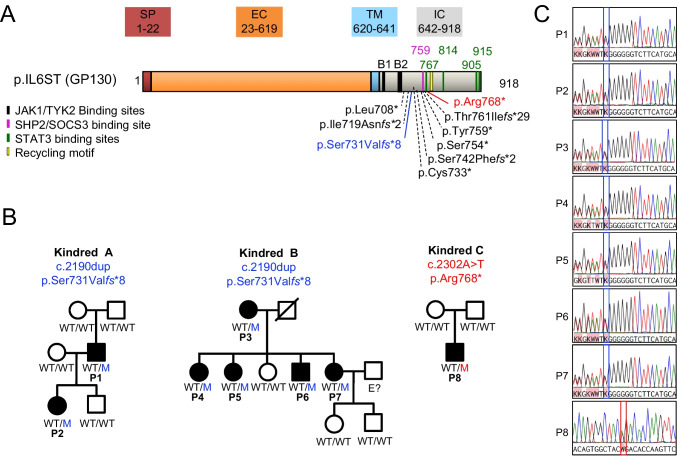
*IL6ST* variants. **A** Schematic representation of GP130 and population genetics of *IL6ST* alleles. The p.(Leu708*), p.(Ile719Asnfs*2), p.(Cys733*), p.(Ser742Phefs*2), p.(Ser754*), p.(Tyr759*), and p.(Thr761Ilefs*29) variants were previously reported to be LOF [6]. The new mutants described here are shown in red and blue. EC, extracellular domain; IC, intracellular domain; SP, signal peptide; TM, transmembrane domain. **B** Pedigrees showing the familial segregation of the c.2190dupG (p.(Ser731Valfs*8)) and c.2302A > T (p.(Arg768*)) mutant *IL6ST* alleles. Individuals of unknown genotype are labeled “E?” **C** Chromatograms of the IL-6ST variants found in P1, P2, P3, P4, P5, P6, P7, and P8

A new pathogenic variant of the *IL6ST* gene was identified in P1, P2, P3, P4, P5, P6, and P7 by the targeted sequencing of 84 genes implicated in monogenic human inborn errors of innate immunity, or Sanger sequencing. This variant, c.2190dupG (p.(Ser731Valfs*8)), affects residues C-terminal to the JAK1/TYK2 binding sites and N-terminal to the recycling motif, the four STAT3 binding residues, and the SHP2/SOCS3 binding site (Fig. 2A). The c.2190dupG (p.(Ser731Valfs*8)) variant is not reported in common population databases (gnomAD v2.1 and ClinVar) [22]. It occurred de novo in P1, who is the father of P2 (Fig. 2B). The same variant is present in P3, and four of her children — P4, P5, P6, and P7 — suggesting that c.2190dupG occurs in a mutational hotspot, probably due to a stretch of six consecutive guanine residues. None of the other members of the family tested carried the *IL6ST* variant in their genome (Fig. 2B). Chromatograms showing the c.2190dupG variant identified in the patients are shown in Fig. 2C.

WES was performed on P8 and revealed a previously unknown monoallelic variant of *IL6ST*, c.2302A > T; p.(Arg768*), predicted to result in a premature stop codon within the intracellular domain of GP130 (Fig. 2A). The c.2302A > T (p.(Arg768*)) variant, confirmed by Sanger sequencing (Fig. 2C), is not present in common population databases (gnomAD v2.1 and ClinVar). It was found C-terminal to the transmembrane domain and was predicted to result in a premature stop codon C-terminal to the SHP2/SOCS3 binding motif (Tyr759) and the first of the four STAT3 binding residues (Tyr767), but N-terminal to the recycling motif (Ser782-Leu287) (Fig. 2A). Parental segregation analysis showed that this variant had occurred de novo in the proband (Fig. 2B). This variant is more C-terminal than any of the AD HIES–causing GP130 variants characterized to date [6, 23]. Nonsense-mediated mRNA decay was not suspected for either of the variants (p.(Ser731Valfs*8) or p.(Arg768*)), because premature termination codons occurring 50–55 nucleotides before the last exon:exon junction are not constrained by this process [24, 25]. As a result, *IL6ST* mRNA levels were predicted to be unaffected by the premature termination codons occurring in the last exon.

### The p.(Arg768*) GP130 Variant Is Overexpressed at the Surface of HEK293T Cells, Whereas the p.(Ser731Valfs*8) Variant Is Not

**Fig. 3:**
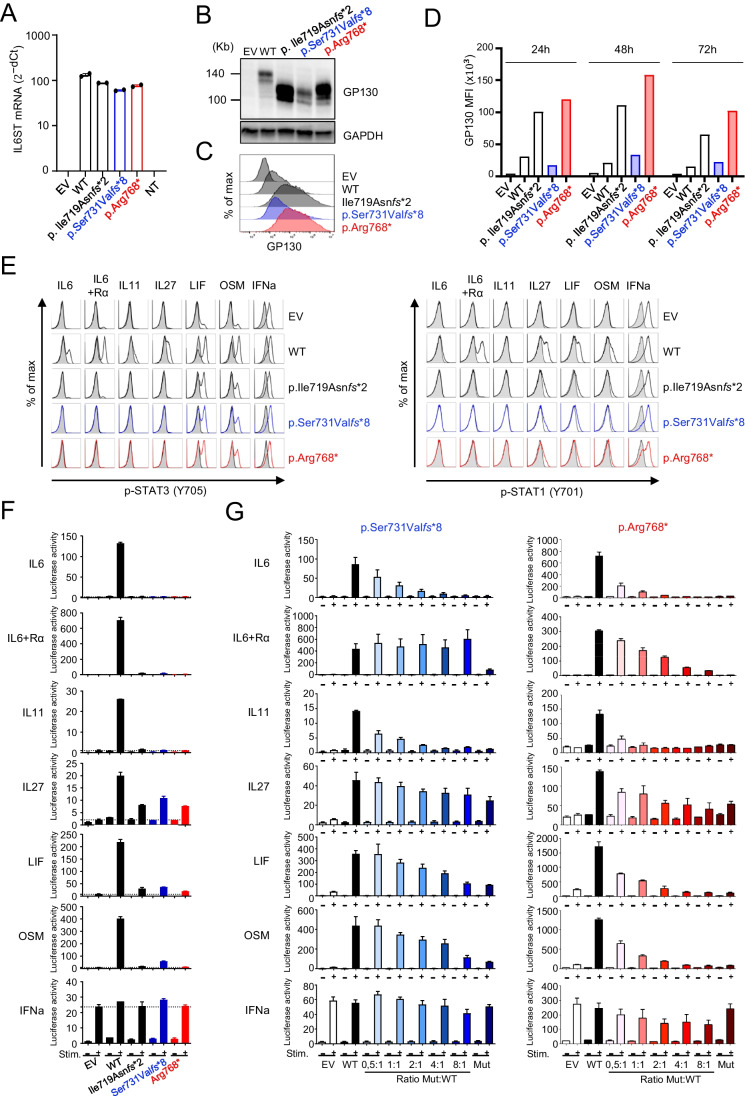
Functional analysis of the p.(Ser731Valfs*8) and p.(Arg768*) GP130 variants in GP130-deficient HEK293T cells transfected with an empty pCMV6 plasmid (EV) or with pCMV6 plasmids encoding the WT GP130 or the p.(Ile719Asnfs*2), p.(Ser731Valfs*8), or p.(Arg768*) GP130 variant. **A** RNA was extracted 48 h after transfection and subjected to RT-qPCR for GP130. Data are presented as 2^−ΔCt^ values after normalization against GUS (endogenous control) expression (ΔCt). Results representative of three independent experiments are shown. **B** Western blot analysis of GP130 expression 48 h after transfection (cropped images of blots hybridized with anti-GP130 antibody and re-blotted with anti-GAPDH antibody for loading control). **C** Flow cytometry analysis of GP130 at the cell surface 48 h after transfection. **D** MFI reflecting GP130 cell surface expression 24 h, 48 h, and 72 h after transfection. **E** Flow cytometry analysis of the phosphorylation of STAT3 and STAT1 after the stimulation of cells with IL-6, IL-6 + IL-6Rα, IL-11, IL-27, LIF, OSM, and IFN-α as a control. **F** Analysis of STAT3 transcriptional activity with luciferase reporter assays on transfected GP130-deficient HEK293T cells stimulated with the cytokine indicated (+) or left unstimulated (−). Horizontal dotted lines indicate the luciferase activity after stimulation with the cytokine indicated, in cells transfected with the empty pCMV6 vector. The results shown are the mean and standard error for a technical triplicate. **G** GP130-deficient HEK293T cells were transfected with various proportions of vectors encoding the p.(Ser731Valfs*8) or p.(Arg768*) GP130 mutants to highlight the DN effect of the variants. Empty vector with the same backbone as the GP130 constructs (WT and variants) was added to ensure that the same amount of DNA was added for each transfection ratio studied. Representative luciferase assay results for three independent experiments are shown

GP130-deficient HEK293T cells were transfected with pCMV6 plasmids encoding the WT GP130, the p.(Ser731Val*f*s*8) variant or the p.(Arg768*) variant, the previously described p.(Ile719Asnfs*2) variant (lacking the recycling motif, the SHP2/SOCS3-binding site, and all the STAT3-binding sites (Fig. 2A)) [6], or an empty vector (EV). After 48 h of transfection, *IL6ST* mRNA levels were evaluated by RT-qPCR, which showed that expression levels were similar in cells expressing the wild-type form and in cells expressing the variants (Fig. 3A). At the same time point, western blotting and immunodetection with a monoclonal anti-GP130 antibody (clone E8, recognizing amino acids 365–619) revealed the presence of WT proteins of ∼140 kDa and truncated proteins for the p.(Ser731Valfs*8), p.(Arg768*), and p.(Ile719Asnfs*2) variants (Fig. 3B). The p.(Arg768*) and p.(Ile719Asnfs*2) variants were both more strongly expressed than the WT. By contrast, the p.(Ser731Valfs*8) variant was expressed at a level similar to that for the WT allele (Fig. 3B). After 48 h of transfection, the surface expression of the GP130 variants was also analyzed by flow cytometry (Fig. 3C, D). GP130-deficient HEK293T cells transfected with the EV remained GP130-negative, whereas cells transfected with the WT GP130 construct clearly expressed GP130. As expected from the western blot and prior observations [6], the p.(Arg768*) and p.(Ile719Asnfs*2) variant proteins were more strongly expressed at the cell surface than the WT protein. Surprisingly, but consistent with the western blot data, the p.(Ser731Val*f*s*8) variant did not accumulate at the cell surface despite the absence of the canonical recycling motif (Ser782-Leu787), and was instead found at levels similar to those for the WT GP130 (Fig. 3C, D). By contrast, like all previously reported HIES-associated truncated variants, the p.(Arg768*) variant was found to encode a receptor chain that reaches the cell surface where it accumulates [6, 23].

### The p. (Ser731Valfs*8) and p.(Arg768*) GP130 Variants Impair STAT3 Activation

The phosphorylation of STAT3 and STAT1 was assessed by flow cytometry in GP130-deficient HEK293T cells transfected with the EV, and with vectors expressing either the WT control GP130, or the p.(Ile719Asnfs*2), p.(Ser731Val*f*s*8), or p.(Arg768*) GP130 mutants, after stimulation with IL-6, IL-6 + IL-6Rα, IL-11, IL-27, LIF, OSM, or IFN-α as a control (Fig. 3E). Following stimulation with IL-6, IL-6 + IL-6Rα, or IL-11, the p.(Ile719Asnfs*2), p.(Ser731Val*f*s*8), and p.(Arg768*) mutants failed to phosphorylate STAT3 and STAT1. Stimulation with IL-27 resulted in no STAT3 phosphorylation, but STAT1 was weakly phosphorylated in cells transfected with any of the three mutants. Stimulation with LIF and OSM resulted in weak STAT3 and STAT1 phosphorylation in cells transfected with any of the three mutants. The phosphorylation of STAT1 or STAT3 after stimulation with IL-27, LIF, or OSM probably occurs due to the intrinsic signaling capacity of IL-27RA, LIF-R and OSM-R [26–29]. These data are consistent with published data for HIES-associated truncating GP130 variants in similar settings [6].

The ability of the variants to activate STAT3 signaling was further assessed in a luciferase reporter assay, as previously described [6]. GP130-deficient HEK293T cells were co-transfected with an empty pCMV6 vector (EV), or a plasmid either encoding the WT or one of the GP130 variants, and a STAT3 reporter plasmid carrying the luciferase cDNA. Cells were left unstimulated or were stimulated with IL-6 family cytokines or IFN-α as a control. Luciferase activity was then measured in the transfected cells. As expected, stimulation with IL-6, IL-6 + IL-6Rα, IL-11, IL-27, LIF, or OSM induced strong luciferase activity in the WT GP130-expressing cells. By contrast, there was very little or no luciferase activity in stimulated cells expressing the p.(Ile719Asnfs*2), p.(Ser731Val*f*s*8), or p.(Arg768*) GP130 variants. Stimulation with IFN-α induced strong luciferase activity in all samples (Fig. 3F). Thus, the newly identified p.(Ser731Val*f*s*8) and p.(Arg768*) GP130 variants are impaired in the ability to mediate STAT3 activation, consistent with other previously reported variants [6].

### The p.(Ser731Valfs*8) and p.(Arg768*) GP130 Variants Have DN Effects

We then used the same luciferase reporter assay to test the hypothesis that, like the previously described variants affecting the integrity of the intracellular GP130 region [6], the p.(Ser731Val*f*s*8) and p.(Arg768*) GP130 variants exerted DN effects on the WT GP130. GP130-deficient HEK293T cells were transfected with a plasmid encoding the WT GP130 construct in the presence of various proportions of a plasmid encoding a GP130 variant. Variable amounts of empty vector were added to each well, to ensure that the same total amount of DNA was present during transfection. The activation of STAT3 transcriptional activity was assessed by measuring the luciferase signal after stimulation with the indicated cytokines of the IL-6 family (Fig. 3G). IFN-α stimulation was used as a positive control. Both GP130 variants exerted strong DN effects on the WT allele at a variant:WT ratio of 1:1, after stimulation with IL-6 or IL-11. As previously reported for other variants [6], the two new variants had weaker DN effects on LIF and OSM signaling, with only incomplete effects on IL-27 signaling (Fig. 3G). Together with the intrinsic signaling capacities of IL-27RA, LIF-R, and OSM-R [26–29], the trimeric nature of the cell surface complexes formed by LIF, OSM, and IL-27 with GP130 and their coreceptor chains may underlie their lower sensitivity to DN effects at physiological variant:WT ratios (e.g., 0.5:1, 1:1). By contrast, IL-6 and IL-11 form hexameric complexes with their coreceptors and two molecules of GP130, and are highly affected by the DN effect at physiological variant:WT ratios [6]. The p.(Arg768*) variant consistently had a stronger DN effect than the p.(Ser731Val*f*s*8) variant, probably reflecting the normal level of expression of the latter at the cell surface. Indeed, this mutant had no impact on IL-6/IL-6Rα trans-signaling in the conditions tested, and supraphysiological amounts (ratios of 2:1, 4:1, 8:1) of the variant protein were required for the detection of a visible effect on IL-27, LIF, and OSM signaling. The lack of impact on IL-6/IL-6Rα trans-signaling is not surprising. Indeed, this readout is always the least affected in assays of dominant-negative effects, probably because trans-signaling is dependent on GP130 solely for signal transduction, and is therefore affected only by cytokine availability [6]. Overall, our data show that both the p.(Ser731Valfs*8) and p.(Arg768*) variants display dominant-negative activity, but with a less potent effect for the p.(Ser731Valfs*8) variant, reflecting its normal levels of expression at the cell surface.

### The p.(Ser731Val*f*s*8) Variant Does Not Accumulate in Large Amounts at the Surface of Primary Cells

**Fig. 4:**
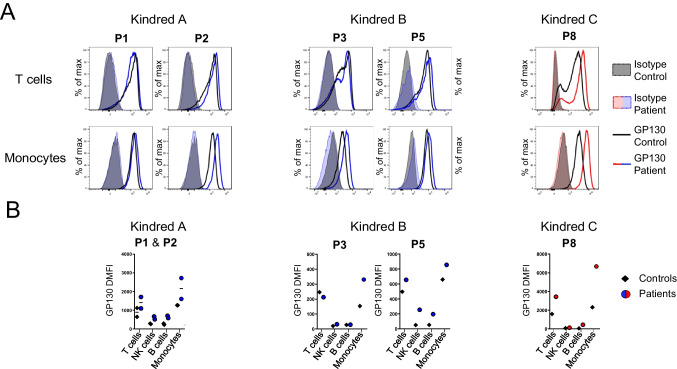
GP130 expression in the patients’ primary lymphocytes and monocytes**.** Patients carrying the p.(Ser731Valfs*8) allele are represented in blue, and P8, carrier of the p.(Arg768*) variant, is shown in red. **A** Representative flow cytometry histogram of GP130 levels (black or colored lines) in T cells and monocytes from patients and controls relative to isotype (colored area). **B** The graphs show the ΔMFI of GP130 measured by flow cytometry in primary T cells, B cells, NK cells, and monocytes from controls and P1, P2, P3, P5, and P8

We evaluated the impact of the variants on endogenous GP130 expression. We measured GP130 fluorescence intensity by flow cytometry at the cell surface of fresh blood mononuclear cells from P1, P2, P3, P5, and P8 (Fig. 4) and healthy controls. GP130 was strongly expressed in T lymphocytes and monocytes, but poorly expressed in B cells and NK cells from controls. The cells from P8, who carried the p.(Arg768*) variant, displayed clear cell surface GP130 accumulation relative to controls. This accumulation was particularly visible in monocytes, and corroborated our previous findings [6]. By contrast, consistent with the in vitro expression data (Fig. 3), the cells, including monocytes, from patients carrying the p. (Ser731Valfs*8) variant presented normal or low levels of GP130 accumulation at the cell surface relative to controls (Fig. 4). Overall, our data from primary cells from the patients confirm that the p.(Arg768*) variant results in strong GP130 accumulation at the cell surface, whereas the p.(Ser731Valfs*8) variant results in normal or slightly higher levels of GP130 expression at the cell surface.

### Immunological Abnormalities Correlate with GP130 Accumulation at the Cell Surface

**Fig. 5:**
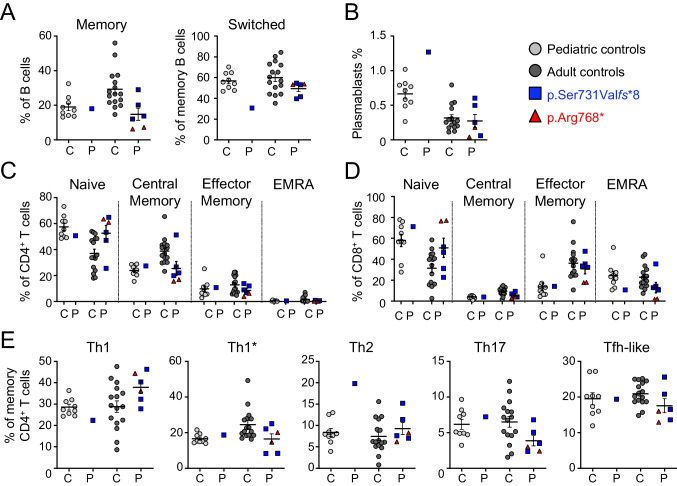
Leukocyte immunophenotyping.** A** Frequency of total memory B cells (CD19^+^CD27^+^) within the B cell compartment and frequency of switched (CD19^+^CD27^+^IgM^−^) memory cells within the memory B cell compartment. **B** Frequency of plasmablast cells (CD19^+^CD20^−^) within the B cell compartment. **C**,** D** Frequency of naive (CD45RA^+^CCR7^+^), central memory (CD45RA^−^CCR7^+^), effector memory (CD45RA^−^CCR7^−^), and T_EMRA_ (CD45RA^+^CCR7^−^) cells among CD4^+^ (**C**) and CD8^+^ (**D**) T cells. **E** Frequency of the T helper subsets indicated within the CD4^+^ memory compartment. Subsets were defined as follows: Th1 (CXCR5^−^CXCR3^+^CCR4^−^CCR6^−^), Th1* (CXCR5^−^CXCR3^+^CCR4^−^CCR6^+^), Th2 (CXCR5^−^CXCR3^−^CCR4^+^CCR6^−^), Th17 (CXCR5^−^CXCR3^−^CCR4^+^CCR6^+^), and Tfh (CXCR5^+^). **A**–**E** Phenotyping was performed in pediatric controls (*n* = 9, in gray), adult controls (*n* = 16, in dark gray), and patients with AD *IL6ST* mutations (*n* = 6, in blue and red). Horizontal lines represent the mean and vertical lines represent the standard error. P1 (38 years old), P2 (11 years old), P4 (47 years old), P5 (45 years old), and P6 (33 years old), all of whom carry the p.(Ser731Valfs*8) variant, are represented by blue squares. P8 (20 years old), carrier of the p.(Arg768*) variant, is represented by red triangles

Leukocyte immunophenotyping was performed by mass cytometry (CyTOF) for five patients carrying the p.(Ser731Valfs*8) allele (P1, P2, P4, P5, and P6) and the patient carrying the p.(Arg768*) allele (P8) (Fig. 5; Figure S1). Samples from 16 healthy adults were used as controls for P1, P4, P5, P7, and P8, and samples from nine healthy children were used as controls for P2. Patients carrying the p.(Ser731Valfs*8) variant that does not accumulate at the cell surface had normal immunophenotyping results, similar to those for age-matched controls, except that plasmablast and Th2 cell levels were high in P2. By contrast, the patient carrying the p.(Arg768*) variant (P8) had an abnormally low frequency of memory B cells, high levels of naïve and low levels of central memory CD4 and CD8 T cells, a Tfh frequency in the lower part of the normal range for controls, and normal Th subset proportions among memory cells. Except for the normal frequency of Th2 cells, the immunophenotype of P8 was consistent with our previous report [6]. However, it should be noted that, at the time of immunophenotyping, P8 was receiving dupilumab treatment, which may have modified the Th2-related values [30, 31]. Overall, these data suggest that the observed immunological abnormalities are correlated with the accumulation of DN forms of GP130 at the cell surface.

## Discussion

In this report, identifying eight additional patients, we expand the genetic landscape of AD HIES caused by DN *IL6ST* variants. We described the GP130 p.(Ser731Valfs*8) variant in two unrelated families. This variant provides an additional example of genetic defects affecting the intracellular portion of GP130 N-terminal to the SHP2/SOCS3 binding site and STAT3 binding motifs, in a region potentially corresponding to a causal variant hotspot for *IL6ST*. Surprisingly, this variant did not accumulate at the cell surface despite lacking the recycling motif. It remains unclear whether the additional eight-amino acid stretch generated by the p.(Ser731Valfs*8) frameshift variant reduces the accumulation of the variant GP130 by decreasing the stability of the protein, or by other, as yet unknown, mechanisms. We also identified the p.(Arg768*) variant, the only variant retaining the SHP2/SOCS3 binding site and the first STAT3 binding residue, but lacking the GP130 recycling motif identified to date (Fig. 2A).

The clinical phenotype of the patients was largely similar to that previously reported [6], with recurrent and severe bacterial and mycobacterial infections of the respiratory tract, atopy, and variable extra-hematopoietic features, such as scoliosis, joint hyperextension, deciduous tooth retention, and typical facies. Biological features included high IgE levels and eosinophilia. However, consistent with the lower levels of the GP130 variant at the cell surface, the patients carrying the p.(Ser731Valfs*8) variant had a milder overall phenotype, with a median HIES score of 26 (range: 12–42), versus a median of 44 (range: 20–70) for the other reported patients. The subjects carrying the p.(Ser731Valfs*8) variant nevertheless displayed a high level of clinical heterogeneity, ranging from almost asymptomatic (P7) to severe HIES (P5). We previously reported that patients had high frequencies of naïve CD4 and CD8 T cells, and Th2 cells, and low frequencies of memory B cells and Tfh-like cells [6]. The immunophenotyping of P8, who carries the p.(Arg768*) variant that accumulates in large amounts at the cell surface, was consistent with this previous report, except for the normal frequency of Th2 cells, which may be explained by the patient’s dupilumab treatment. By contrast, the patients carrying the p.(Ser731Valfs*8) variant that is not overexpressed at the cell surface had a largely normal immunophenotype. This observation suggests that immunophenotyping results may correlate with the degree of DN GP130 variant accumulation at the cell surface. We also previously reported that patients had normal acute-phase reactions during infections [6]. Here, P8 clearly had weak inflammatory responses during infections, suggesting that patients with DN HIES may also display variability in terms of the ability to mount acute-phase reactions.

In our original report [6], we described one DN HIES kindred with a milder clinical presentation (NIH scores between 20 and 29) than the other families identified (NIH scores between 37 and 70). We speculated that this milder phenotype might be due to the p.(Thr761Ilefs*29) variant carried by the family members. Indeed, at the time of our first report, p.(Thr761Ilefs*29) was the most C-terminal variant associated with HIES and the only one predicted to retain the SHP2/SOCS3 binding site (Tyr759) while lacking the recycling motif (Ser782-Leu787) and all the STAT3 binding sites. The p.(Arg768*) variant identified in P8 is located even closer to the C-terminus than p.(Thr761Ilefs*29) and is predicted to retain both the SHP2/SOCS3 binding site (Tyr759) and the first STAT3 binding residue (Tyr767), while lacking the recycling motif (Ser782-Leu787) (Fig. 2A). Like the p.(Thr761Ilefs*29) variant, the p.(Arg768*) variant accumulates at the cell surface and is unable to mediate the phosphorylation of STAT1 and STAT3 or the activation of STAT3 signaling, despite the conservation of one STAT3 binding site. However, unlike the patients carrying the p.(Thr761Ilefs*29) variant, P8 had a very severe, life-threatening HIES phenotype, with a NIH HIES score of 51. Contrary to our previous hypothesis, this observation demonstrates that the retention of the SHP2/SOCS3 binding site is not necessarily associated with a milder HIES phenotype.

Based on the severity of the clinical presentation in P8 and the functional characterization of the p.(Arg768*) mutant, it is tempting to conclude that the first STAT3 binding residue Tyr767 does not play a crucial role in maintaining physiological levels of STAT3 signaling and, thus, in protecting against the development of a HIES phenotype. However, the p.(Arg768*) truncation would probably affect the stability of STAT3 binding to residue Tyr767, thereby countering the activation of some residual STAT3-mediated signaling. This hypothesis appears more likely according to population genetics. Relative to p.(Arg768*), the p.(Ser789*) variant identified in controls from the gnomAD V2.1 database conserves only the first STAT3 binding residue (Tyr767), but retains the recycling motif [6]. Despite the predicted retention of one STAT3 binding site by both the p.(Arg768*) and p.(Ser789*) variants, only the p.(Ser789*) variant conserves some ability to activate STAT3 signaling in vitro. It is unknown whether individuals carrying the p.(Ser789*) variant, or, more generally, variants truncated between the recycling motif and the second STAT3 binding motif, can develop mild clinical and biological presentations of HIES (e.g., allergies, high IgE levels). It is tempting to speculate that the severe HIES phenotype develops only in individuals with variants N-terminal to the recycling motif, promoting the accumulation of the DN protein at the cell surface. However, the discovery of HIES patients carrying the p.(Ser731Valfs*8) variant challenges this hypothesis. Indeed, despite lacking all the STAT3 binding sites and the recycling motif, the DN p.(Ser731Valfs*8) variant did not accumulate in large amounts at the cell surface and was associated with heterogeneous clinical presentations, ranging from mild to severe. Data concerning the clinical and biological features of more patients with variants of *IL6ST* resulting in a truncation between the first and the second STAT3 binding site are therefore required to address this issue.

In summary, we report here two new DN variants of *IL6ST*. The p.(Ser731Valfs*8) variant lacks the recycling motif, but does not accumulate at the cell surface. It generally causes milder forms of HIES, albeit with considerable clinical heterogeneity among carriers. The p.(Arg768*) variant is the most C-terminal variant reported to date in patients. It causes a severe HIES phenotype despite the conservation of the SHP2/SOCS3 binding site and the first STAT3 binding site. These observations highlight the need for further investigations of HIES-causing genetic defects and of the specific pathophysiologic mechanisms leading to disease.

## Supplementary Information

Below is the link to the electronic supplementary material.

Supplementary file1 (DOCX 280 KB)

## Data Availability

Data and reagents are available on request from the authors.
