# Correction to: New Dominant‑Negative IL6ST Variants Expand the Immunological and Clinical Spectrum of GP130‑Dependent Hyper‑IgE Syndrome

**DOI:** 10.1007/s10875-023-01539-y

**Published:** 2023-06-21

**Authors:** Tiphaine Arlabosse, Marie Materna, Orbicia Riccio, Caroline Schnider, Federica Angelini, Matthieu Perreau, Isabelle Rochat, Andrea Superti-Furga, Belinda Campos-Xavier, Sébastien Héritier, Anaïs Pereira, Caroline Deswarte, Romain Lévy, Marco Distefano, Jacinta Bustamante, Marie Roelens, Raphaël Borie, Mathilde Le Brun, Bruno Crestani, Jean‑Laurent Casanova, Anne Puel, Michaël Hofer, Claire Fieschi, Katerina Theodoropoulou, Vivien Béziat, Fabio Candotti

**Affiliations:** 1 Pediatric Immuno‑Rheumatology of Western Switzerland, Pediatrics Service, Women‑Mother‑Child Department, Lausanne University Hospital, Lausanne, Switzerland; 2 Laboratory of Human Genetics of Infectious Diseases, Necker Branch, Institut National de La Sante Et de La Recherche Medicale (INSERM), U1163 Paris, France; 3 Paris Cite University, Imagine Institute, Paris, France; 4 Division of Immunology and Allergy, Lausanne University Hospital and University of Lausanne, Lausanne, Switzerland; 5 Pediatric Pulmonology and Cystic Fibrosis Unit, Pediatrics Service, Women‑Mother‑Child Department, Lausanne University Hospital, Lausanne, Switzerland; 6 Division of Genetic Medicine, Lausanne University Hospital and University of Lausanne, Lausanne, Switzerland; 7 Division of Pediatric Hematology and Oncology, Armand Trousseau Hospital, Sorbonne University, Paris, France; 8 St. Giles Laboratory of Human Genetics of Infectious Diseases, Rockefeller Branch, The Rockefeller University, New York, NY USA; 9 Study Center for Primary Immunodeficiencies, AP-HP, Necker Children Hospital, Paris, France; 10 Department of Medicine, Bichat Hospital, AP-HP, Paris, France; 11 Department of Pulmonology A, Reference Center for Rare Pulmonary Diseases, Bichat Hospital, AP-HP, Paris, France; 12 Department of Pediatrics, Necker Hospital for Sick Children, AP-HP, 75015 Paris, France; 13 Howard Hughes Medical Institute, The Rockefeller University, New York, NY 10065 USA; 14 Department of Clinical Immunology, Paris Cite University, Assistance Publique Hopitaux de Paris (AP-HP), Saint-Louis Hospital, Paris, France

**Correction to: Journal of Clinical Immunology**

10.1007/s10875-023-01517-4

Due to typesetting mistake, the bottom panel of Fig. 3B (GAPDH) was left blank rather than showing the control bands.

The original version has been corrected.

