# Supplementary material for: New Dominant-Negative IL6ST Variants Expand the Immunological and Clinical Spectrum of GP130-Dependent Hyper-IgE Syndrome
Source: J Clin Immunol. 2023 Jun 5;43(7):1566–80. doi: 10.1007/s10875-023-01517-4 (PMC10499999; doi:10.1007/s10875-023-01517-4)

**Table S1** – Antibodies used for PBMC staining

| **Fluorochrome** | **Target** | **Clone** | **Manufacturer** | **Catalog** |
| --- | --- | --- | --- | --- |
| **PE** | GP130 / isotype IgG1 | AM64 / MOPC-21 | BD / BD | 555757 / 559320 |
| **APC-CY7** | CD19 | SJ25C1 | BD | 557791 |
| **FITC** | CD14 | M5E2 | BD | 555397 |
| **PE-CY7** | CD56 | N901 | Beckman Coulter | A21692 |
| **BV421** | CD27 | O323 | Biolegend | 302824 |
| **APC** | CD3 | UCHT1 | BD | 555335 |

**Table S2 –** Antibodies used for mass cytometry on fresh whole blood

| **Metal** | **Target** | **Clone** | **Manufacturer** | **Catalog** |
| --- | --- | --- | --- | --- |
| **163Dy** | CXCR3 | G025H7 | Fluidigm | 3163004B |
| **152Sm** | TCRgd | 11F2 | Fluidigm | 3152008B |
| **142Nd** | CD19 | HIB19 | Fluidigm | 3142001B |
| **144Nd** | CD38 | HIT2 | Fluidigm | 3144014B |
| **151Eu** | CD123 | 6H6 | Fluidigm | 3151001B |
| **153Eu** | Va7.2 | 3C10 | Fluidigm | 3153024B |
| **154Sm** | CD3 | UCHT1 | Fluidigm | 3154003B |
| **155Gd** | CD45RA | HI100 | Fluidigm | 3155011B |
| **158Gd** | CD27 | L128 | Fluidigm | 3158010B |
| **159Tb** | CD1c | L161 | Biolegend | 331502 |
| **161Dy** | CLEC9A | 8F9 | Fluidigm | 3161018B |
| **164Dy** | CD161 | HP-3G10 | Fluidigm | 3164009B |
| **168Er** | CD8 | SK1 | Fluidigm | 3168002B |
| **170Er** | iNKT | 6B11 | Fluidigm | 3170015B |
| **175Lu** | CCR4 | L291H4 | Fluidigm | 3175035A |
| **174Yb** | CD4 | RPA-T4 | Biolegend | 300502 |
| **162Dy** | CD21 | REA940 | Miltenyi Biotec Inc. | 130-124-315 |
| **165Ho** | NKG2C | REA205 | Miltenyi Biotec Inc. | 130-122-278 |
| **148Nd** | CD20 | 2H7 | Biolegend | 302302 |
| **173Yb** | HLA-DR | L243 | Fluidigm | 3173005B |
| **156Gd** | CCR10 | REA326 | Miltenyi Biotec Inc. | 130-122-317 |
| **089Y** | CD45 | HI30 | Fluidigm | 3089003B |
| **116Cd** | CD66b | QA17A51 | Biolegend | 396902 |
| **141Pr** | CCR6 | G034E3 | Fluidigm | 3141003A |
| **143Nd** | CD127 | A019D5 | Fluidigm | 3143012B |
| **147Sm** | CD11c | Bu15 | Fluidigm | 3147008B |
| **149Sm** | CD25 | 2A3 | Fluidigm | 3149010B |
| **150Nd** | NKVFS1 | NKVFS1 | Bio Rad | MCA2243GA |
| **167Er** | CCR7 | G043H7 | Fluidigm | 3167009A |
| **169Tm** | NKG2A | Z199 | Fluidigm | 3169013B |
| **171Yb** | CXCR5 | RF8B2 | Fluidigm | 3171014B |
| **166Er** | CD24 | ML5 | Fluidigm | 3166007B |
| **145Nd** | CD31 | WM59 | Fluidigm | 3145004B |
| **160Gd** | CD14 | M5E2 | Fluidigm | 3160001B |
| **176Yb** | CD56 | NCAM16.2 | Fluidigm | 3176008B |
| **172Yb** | CD57 | HNK-1 | Biolegend | 359602 |
| **150Nd** | KIR3DL1L2 | REA970 | Miltenyi Biotec Inc. | 130-126-489 |
| **146Nd** | IgD | IA6-2 | Fluidigm | 3146005B |
| **209Bi** | CD16 | 3G8 | Fluidigm | 3209002B |

Figure S1: Leukocyte immunophenotyping. (A) Frequency of monocyte subsets (CD14hiCD16-, CD14hiCD16+ and CD14lowCD16+). (B) Frequency of the dendritic cell subsets among peripheral blood mononuclear cells (PBMCs). cDC1: conventional type 1 dendritic cells (Lin-HLA-DR+CD11c+CD1c+CD141-), cDC2: conventional type 2 dendritic cells (Lin-HLA-DR+CD11c+CD1c-CD141+), and pDCs: plasmacytoid dendritic cells (Lin-HLA-DR+CD11c-CD123+). (C) Frequency of naïve, transitional and CD21lowCD38- cells within the B-cell compartment. (D) Frequency of non-switched (CD19+CD27+IgM+) cells within the memory B-cell compartment. (E) Frequency of CD56bright cells within the total NK cell compartment (F) Frequency of the indicated NK cell subsets among CD56dim cells. (G) Frequency of the indicated T cell subset within the T-cell compartment, γδ: gamma-delta, MAIT: mucosal-associated invariant T cells (CD161+TCR-V⍺.2+), iNKT: invariant natural killer T cells. (H) Frequency of regulatory T cells (Treg) (CD3+CD4+CD25hiCD127-) and recent thymic immigrants (RTE) within the CD4+ population. (I) Frequency of the granulocyte populations indicated among leukocytes.

**Table S3:** Relevant laboratory values for P8


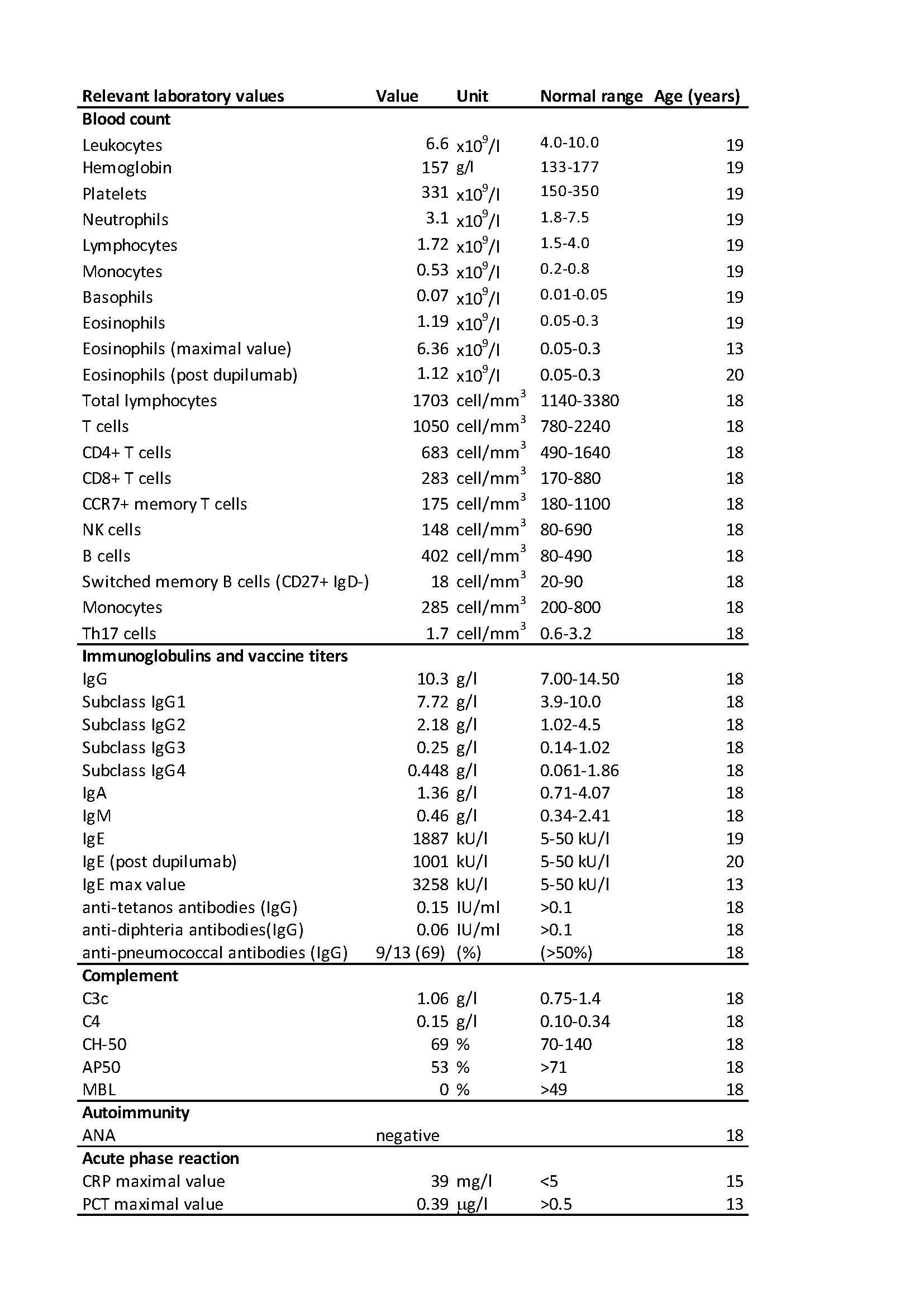

Supplement: Supplementary file 1 — Supplementary file1 (DOCX 280 KB) [file 10875_2023_1517_MOESM1_ESM.docx]
